# Optimal drain position after evacuation of chronic subdural hematomas: a systematic review and network meta-analysis

**DOI:** 10.3389/fneur.2026.1706424

**Published:** 2026-05-12

**Authors:** Ningyu Wei, Hongyu Lu, Yuan Cheng

**Affiliations:** 1 Department of Neurosurgery, Chongqing Medical University Affiliated Second Hospital, Chongqing, China; 2 Department of Rehabilitation Medicine, Chongqing Medical University Affiliated First Hospital, Chongqing, China

**Keywords:** chronic subdural hematoma, drain, network meta-analysis, subdural drain, subgaleal drain, subperiosteal drain, systematic review

## Abstract

**Background:**

Chronic subdural hematoma (CSDH) is one of the most prevalent diseases encountered in neurosurgery. At present, burr-hole hematoma drainage has been established as the standard surgical intervention for CSDH, effectively reducing the risk of postoperative recurrence. The current study employed systematic review and network meta-analysis (NMA) to assess the impact of drain placement in three different anatomical locations—subdural drain (SDD), subperiosteal drain (SPD), and subgaleal drain (SGD)—on treatment outcomes.

**Methods:**

A search was conducted across PubMed, Embase, Cochrane Library, and Web of Science up to February 14, 2026. The Newcastle–Ottawa Scale was used to assess the risk of bias. R (v4.4.0) and Stata18 were used for the NMA.

**Results:**

This NMA included 14 articles comprising 4,161 patients. The drainage locations evaluated were SDD, SPD, and SGD. Pooled results were analyzed based on two classification systems: Classification I (anatomical location) and Classification II (anatomical location + technique). (1) Recurrence rate: Classification I: According to the league table, SGD was associated with a significantly lower recurrence rate versus No_drain [risk ratio (RR) = 0.43, 95% credible interval (CrI): 0.20–0.96]. Based on the surface under the cumulative ranking curve (SUCRA), SGD (78.25%) ranked as the best intervention. Classification II: According to the league table, subgaleal active drainage (SGD_a) was significantly associated with recurrence versus No_drain (RR = 0.26, 95% CrI: 0.10–0.75), and also ranked highest in SUCRA (79.83%). (2) Mortality: Classification I: SGD was associated with reduced mortality (SUCRA = 72.64%). Classification II: subdural irrigation drainage (SDD_irr) showed the best efficacy in reducing mortality (SUCRA = 63.85%).

**Conclusion:**

SGD_a and SDD_irr exhibit significant potential in reducing recurrence rates and mortality, respectively, in the management of CSDH. However, due to the physiological conditions and disease features of old and high-risk populations, careful assessment is necessary when selecting treatment approaches in clinical practice. Further studies should be conducted to clarify the actual efficacy of these two treatment modalities.

**Systematic review registration:**

This study is a systematic review and network meta-analysis and has been registered in the PROSPERO database. Registration ID: CRD42024587692. Official URL: https://www.crd.york.ac.uk/prospero/display_record.php?RecordID=587692.

## 1 Introduction

Chronic subdural hematoma (CSDH), a relatively common condition in neurosurgery, is characterized by the pathological accumulation of blood in the potential space between the dura mater and the arachnoid membrane. The clinical manifestations of CSDH include symptoms such as headache, cognitive dysfunction, and limb weakness. The long-term prognosis of CSDH is generally unfavorable, particularly in older populations (1, 2). According to a 2020 study by Feghali et al. (3), the overall incidence of CSDH is increasing annually, ranging from 1.7 to 20.6 per 100,000 individuals. The median age of onset has increased from 73 to 79 years, and the incidence nearly doubles in individuals aged 80 and above. Based on linear prediction models, the number of CSDH cases is projected to increase by 78.3% by 2040 (1, 4, 5). Currently, the standard treatment for CSDH is primarily burr-hole drainage. This procedure effectively removes hematomas by establishing a closed subdural drainage system. Although it is a widely accepted standard procedure and significantly reduces postoperative mortality (6), patients treated with subdural drain (SDD) still face a 5–33% risk of recurrence (7, 8). Thus, exploring alternative approaches is particularly crucial for lowering the risk of recurrence.

In previous studies regarding drain placement locations, the focus has primarily been on direct comparisons between the following two groups, with varying academic opinions in this field. First, regarding studies on SDD versus subperiosteal drain (SPD), a 2021 randomized controlled trial (RCT) by Pathoumthong et al. (9) and a 2019 multicenter cohort study involving 570 cases both indicated that the drainage outcomes of SDD and SPD were essentially consistent (10, 11). However, Ebel et al. (19) note that while there is no significant difference in recurrence rates between SDD and SPD, drain misplacement is more significant in the SDD group. Comparative studies on SDD versus subgaleal drain (SGD) have yielded the following results. A 2015 study by Oral et al. (12) indicated that both SDD and SGD have high cure rates and low recurrence risks, and that SGD is more suitable for old and high-risk populations. A 2019 study by Häni et al. (13) found no significant impact of SDD and SGD on clinical or radiological outcomes. However, SDD had a relatively higher rate of brain parenchymal injury. A 2022 study by Ozgen et al. (14) demonstrated the effectiveness of SGD_a in reducing the risk of brain parenchymal injury.

Although existing studies have performed comparative analyses of the three drainage modalities (SDD, SPD, and SGD), most studies only compare one or two modalities at a time (15, 16), or group SPD and SGD together due to their similar anatomical hierarchy and compare them with SDD. The three modalities have not been evaluated independently (17). Regarding the overall impact of the three drainage locations on clinical prognosis, especially core indicators of outcome such as postoperative recurrence rates, mortality, and complications, existing studies on ‘differences in the efficacy of drainage locations’ have divergent conclusions (18, 19). It is important to note the anatomical differences between these three drainage methods. In terms of anatomical location, SDD is located within the potential space between the dura mater and the arachnoid membrane. SPD is located between the periosteum and the skull. SGD is located in the loose connective tissue layer between the galea aponeurotica and the periosteum. In terms of anatomical depth, SDD is the deepest, located within the intracranial layer. SPD is in the middle layer, beneath the periosteum. SGD is the most superficial, located within the subcutaneous soft tissue layer. Currently, postoperative drainage techniques for CSDH are mainly classified into three categories. The first is active drainage, which uses negative pressure suction devices with a pressure range of −10 to −20 mmHg to actively aspirate accumulated fluid. The second is irrigation drainage, which involves continuous irrigation with 37 °C warm saline followed by closed drainage. The third is passive drainage, which involves adjusting the patient’s position by elevating the head by 15–30° to achieve natural drainage through gravity and hydrostatic pressure differences (19, 20). Recently, as original research on drainage technology has continued to deepen, related findings have become increasingly abundant. However, from an overall research perspective, studies that comprehensively integrate and analyze different drainage techniques are still insufficient. Therefore, the current study focuses on the characteristics and differences of the three drainage techniques to explore their effects and clinical value.

This systematic review and network meta-analysis (NMA) comprehensively assesses three primary drainage techniques—SDD, SPD, and SGD—by constructing a dual classification system for comparative analysis: Classification I (anatomical location) and Classification II (anatomical location and technical methods). By evaluating the effects of each drainage method on recurrence, mortality, and complications across these classifications, we aim to objectively assess their clinical value. The findings are intended to offer a more targeted and scientific basis for clinical decision-making, assisting clinicians in making more informed choices when selecting drainage methods.

## 2 Materials and methods

This meta-analysis was conducted in accordance with the Preferred Reporting Items for Systematic Reviews and Meta-Analyses statement and its accompanying explanations, which guide reporting systematic reviews evaluating the effects of health interventions (21). It was registered in the International Prospective Register of Systematic Reviews on February 10, 2026 (CRD42024587692).

### 2.1 Literature retrieval

A search was conducted across PubMed, Embase, Cochrane Central Register of Controlled Trials (CENTRAL), and Web of Science for studies on different drainage locations for the treatment of CSDH up to February 14, 2026, with the language limited to English. The search strategy was developed using a combination of subject terms and free words. The search terms included hematoma, subdural, chronic, chronic subdural hematoma, acute subdural hematoma, subepidural hematoma, intracranial subdural hematomas, subdural hemorrhage, drainage, and drain. Details are presented in Supplementary File S1.

### 2.2 Eligibility criteria

The inclusion criteria were developed based on the principle of PICOS (population, intervention, control, outcome, study design) (22).

Population: Patients diagnosed with CSDH.

Intervention: burr-hole hematoma drainage involves three key steps. First, the hematoma is removed using single or double burr-hole craniotomy techniques. This study did not differentiate between the two approaches. However, in clinical practice, a single burr-hole is often preferred due to its lower invasiveness, shorter operative time, and advantages in reducing the risk of complications and shortening hospital stay. Furthermore, the therapeutic effect of single burr-hole is comparable to that of double burr-hole, making it a more common clinical choice (23, 24). Second, drain placement is performed per standard procedures, typically with a dwell time of 24–72 h (25). Finally, one of four drainage methods is selected based on specific clinical needs: no drainage, active drainage, irrigation drainage, or passive drainage. The drain is then accurately placed in different anatomical layers such as SDD, SPD, and SGD.

Comparison: One of the abovementioned drainage locations or no drainage.

Outcome: Recurrence rate, mortality, and complications.

Study design: RCTs, cohort studies.

The following studies were excluded: (1) Duplicate publications, animal experiments, conference abstracts, case reports, letters, guidelines, notes, preliminary reports, registry information, non-English articles, and meta-analyses; (2) Incomplete articles, lack of a control group, no specification of intervention site, and unavailable data.

### 2.3 Literature screening and data extraction

Relevant records from the four databases were imported into EndNote20. Duplicates were removed at first. Inconsistent articles were then excluded by reviewing their titles and abstracts. Eligible studies were finally determined by reading the full texts.

A predefined Excel was used to extract data from the eligible studies. The information encompassed: (1) Basic information: author, year, country, and literature type; (2) Patient information: age, sex, and number of people; (3) Outcome indicators: recurrence rate, mortality, complications, etc.; (4) Relevant data for evaluating the risk of bias.

Two researchers (Ningyu Wei, Hongyu Lu) performed the screening and extracted the data. Any discrepancies were resolved by discussing with a third researcher (Yuan Cheng).

### 2.4 Quality evaluation

The quality of included cohort studies was evaluated using the Newcastle-Ottawa Scale (NOS) (26). The NOS assesses the risk of bias across three domains: selection of study groups, comparability of groups, and ascertainment of either the exposure or outcome of interest. This scale assigns a maximum score of nine, with higher scores indicating higher methodological quality. In this study, we categorized the quality of studies as low (0–3), moderate (4–6), and high (7–9).

The risk of bias in the included RCTs was assessed using the revised Cochrane risk-of-bias tool for randomized trials (RoB 2) (27). RoB 2 covers five specific domains: (1) bias arising from the randomization process, (2) bias due to deviations from intended interventions, (3) bias due to missing outcome data, (4) bias in measurement of the outcome, and (5) bias in selection of the reported result. For each domain, signaling questions were answered to determine the level of the risk of bias, which was categorized as ‘low risk’, ‘some concerns’, or ‘high risk’. The overall risk of bias for each study was determined based on the highest level of risk identified across all domains. A study was classified as ‘low risk’ only if all domains were rated as low risk. A study was classified as ‘some concerns’ if at least one domain raised some concerns, but none were rated as high risk. A study was classified as ‘high risk’ if at least one domain was judged to be at high risk of bias (28). The quality was evaluated by two researchers (Ningyu Wei, Hongyu Lu). Any discrepancies were resolved by discussing with a third researcher (Yuan Cheng).

### 2.5 Statistical analysis

The present research employed R (v4.4.1) and the GeMTC package to construct a model for the NMA. All analyses were performed within a Bayesian framework. The included studies were analyzed using either random-effects or fixed-effects models via the ‘GeMTC’ and ‘rjags’ packages. Posterior distributions of parameters were simulated using Markov chain Monte Carlo methods, a Bayesian approach that provides complete probability distributions for all effect estimates. For each Markov chain, a burn-in phase of 25,000 iterations was executed. Subsequently, 250,000 simulation iterations were performed (with a thinning factor of 10) to generate stable posterior estimates. For fit, comparison, and convergence assessment of the model, the goodness of fit was systematically evaluated to ensure the reliability of the selected model. First, consistency models were compared with inconsistency models, and random-effects variants were compared with fixed-effects variants, by calculating the deviance information criterion (DIC). The model with the lowest value of DIC was selected as the final model for reporting. Second, the leverage and residual contribution of each data point were examined to identify potentially outlying studies that exerted excessive influence on the model or exhibited poor fit. The convergence of the model was comprehensively appraised using the Brooks–Gelman–Rubin diagnostic tool, combined with visual inspection of Markov chain trace plots and posterior density plots. This process ensured that the potential scale reduction factor for all key parameters approximated 1.0 and that the chains demonstrated adequate mixing. For the assessment of inconsistency and calculation of effect size, the consistency model was adopted as the primary analytical framework. When closed loops appeared in the network plot, the node-splitting method was employed to appraise local inconsistency between direct and indirect evidence. Effect sizes were derived from the final Bayesian model and presented as standardized mean differences or mean differences with their 95% credible intervals (CrIs). Results of all pairwise comparisons among interventions were displayed using forest plots and league tables. Statistical significance was determined when 95% CrIs did not contain the value of the null effect. For the ranking of interventions and visualization of the network, probability distributions for the rankings of different interventions were established. The hierarchy of interventions was quantified by calculating the surface under the cumulative ranking curve (SUCRA). SUCRA values ranged from 0 to 100%. Higher values indicated a greater likelihood that a treatment ranked among the best. In addition, a network geometry graph was plotted to visually illustrate the relationships of evidence among different interventions in this analysis. In this graph, the size of nodes was generally proportional to the sample size, and the thickness of edges was proportional to the number of studies supporting that direct comparison.

## 3 Results

The primary goal of the aforementioned studies was to systematically evaluate the impact of different drain placement techniques on the prognosis of CSDH. Accordingly, this study compared the effectiveness of these techniques in reducing postoperative recurrence, mortality, and complications, aiming to provide clinicians with a scientific and reliable basis for selecting the optimal drainage strategy.

### 3.1 Screening of articles

**Figure 1:**
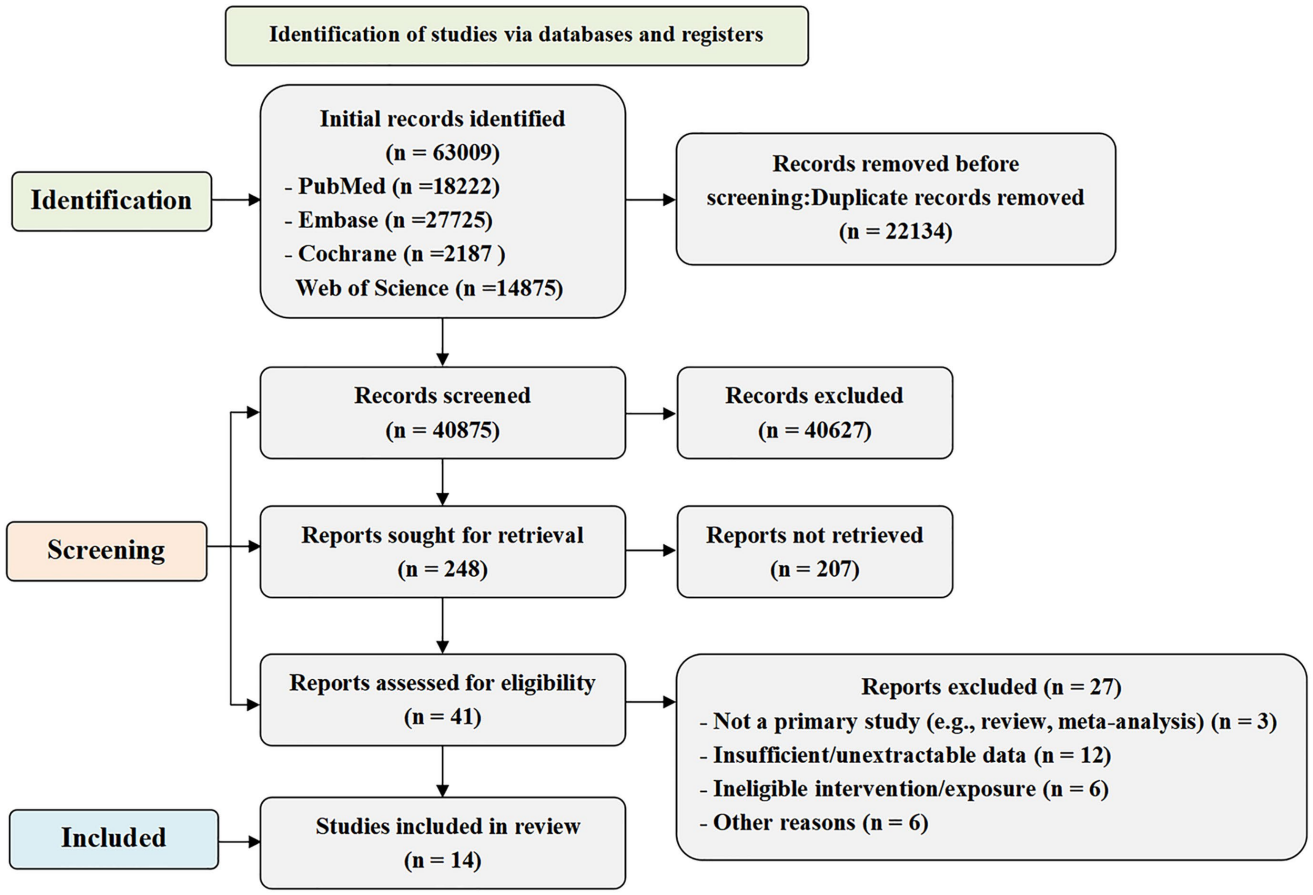
Flowchart of literature screening.

The screening process is illustrated in Figure 1.

The initial search yielded 42,187 articles. First, 14,980 duplicates were removed, leaving 27,207 articles. Subsequently, the titles and abstracts were reviewed, and 27,038 ineligible articles were excluded based on predefined inclusion criteria. The remaining 169 articles were subjected to a full-text review. Eleven articles were removed due to incomplete data or because they were meta-analyses. Ultimately, 14 articles were included (9, 10, 12–14, 16, 20, 29–35).

### 3.2 Characteristics of the included studies

The basic characteristics of the included studies are presented in Supplementary Table S1. A total of 14 studies were included, comprising three RCTs (9, 16, 34) and 11 cohort studies (10, 12–14, 20, 29–33, 35). There were 4,161 patients, with a mean age range of 65.33–81 years. The number of subjects ranged from 42 to 1,260 in these studies. Among the participants, there were 2,414 SDD patients, 412 SPD patients, and 1,040 SGD patients. A total of 295 patients received no_drain or surgery. Two studies were from Turkey (12, 14), two from China (29, 34), one from Malaysia (30), one from Denmark (20), three from the UK (10, 31, 33), two from Switzerland (13, 16), one from Korea (32), one from Thailand (9), and one from Australia (35).

### 3.3 Quality assessment

The NOS was employed to assess the quality of the 11 cohort studies. As detailed in Supplementary Table S2, 6 studies (12–14, 31, 32, 35) achieved a score of 7 (high quality), while 5 studies (10, 20, 29, 30, 33) received a score of 6 (moderate quality). Overall, the cohort studies demonstrated satisfactory methodological quality.

**Figure 2:**
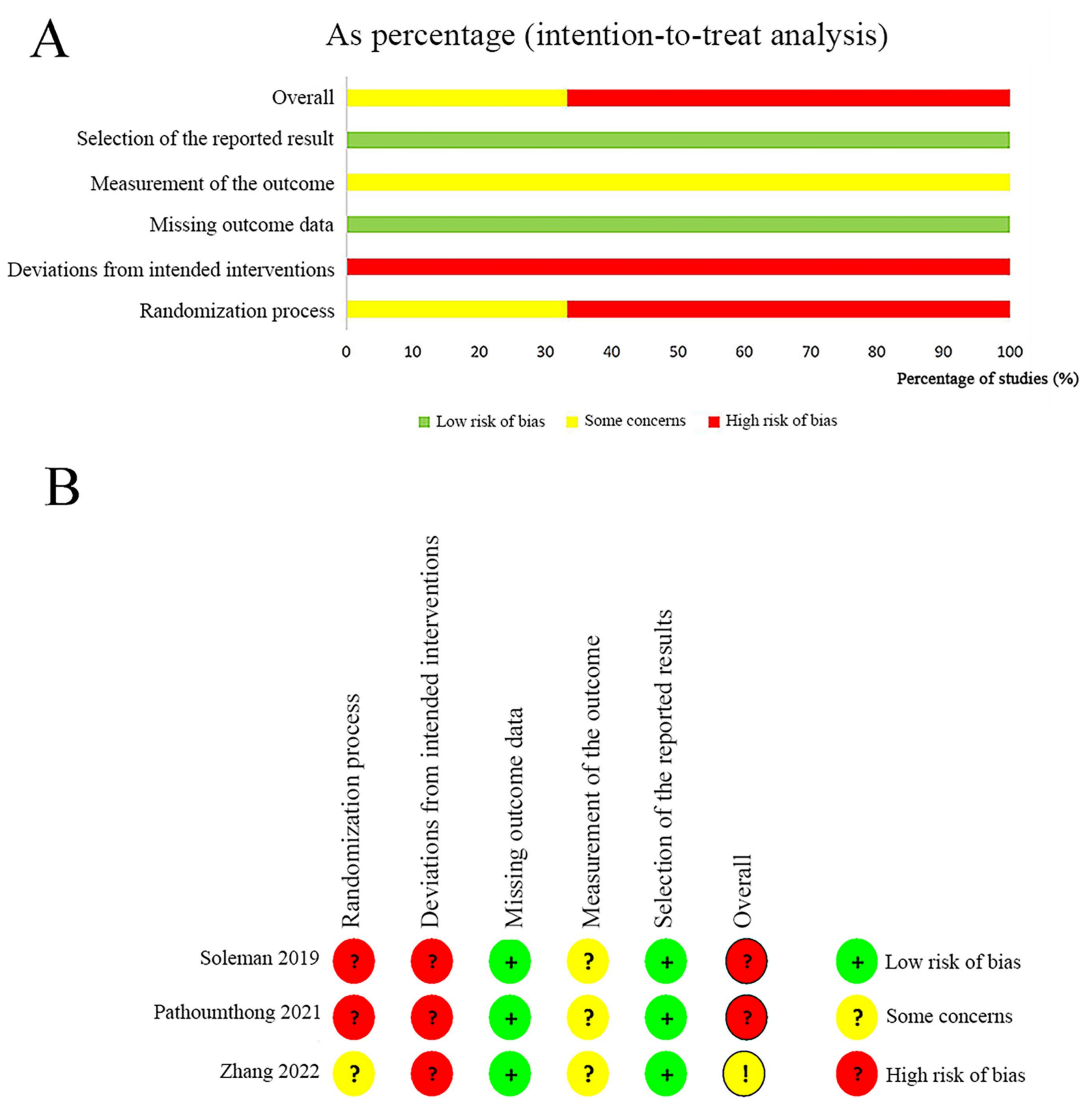
**(A)** As Percentage (ITT); **(B)** ROB 2_IRPG. RoB 2, risk of bias 2 tool; RCT, randomized controlled trial.

RoB 2 was used to evaluate the risk of bias in the 3 RCTs. The overall risk of bias is summarized in Figure 2A. The detailed domain-level assessments are illustrated in Figure 2B. In regard to the five domains, 2 articles were identified as having a high risk, while 1 was classified as raising some concerns. Due to the high risk of bias in the limited number of RCTs, caution is required when interpreting the results derived solely from these trials.

### 3.4 Results

#### 3.4.1 Recurrence

**Figure 3:**
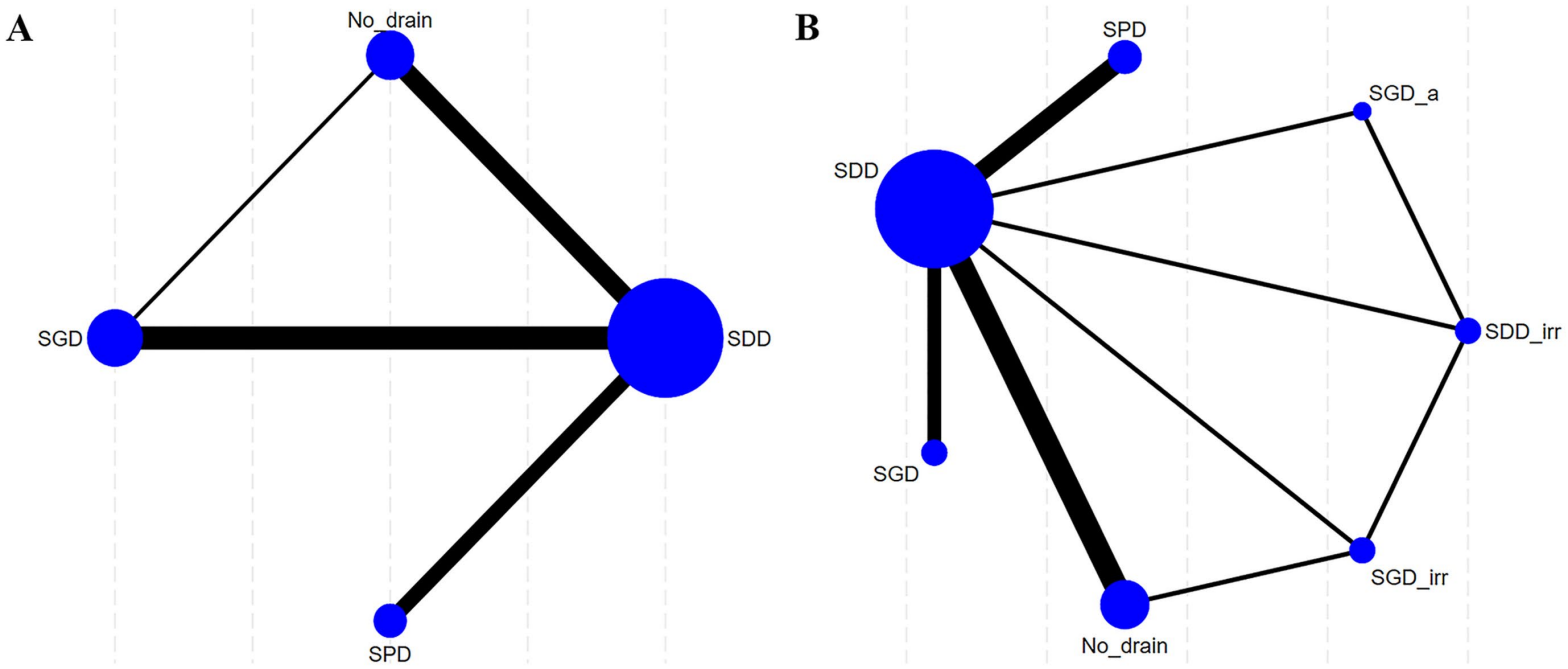
Network plots comparing SDD, SPD, and SGD in relation to recurrence. **(A)** Recurrence in Classification I; **(B)** Recurrence in Classification II. SDD, subdural drain; SDD_irr, subdural irrigation drain; SPD, subperiosteal drain; SGD, subgaleal drain; SGD_irr, subgaleal irrigation drain; SGD_a, Subgaleal active drainage.

Classification I contained four interventions: SDD, SPD, SGD, and No_drain (Figure 3A). Classification II involved seven interventions: SDD, SDD_irr, SPD, SGD, subgaleal irrigation drainage (SGD_irr), SGD_a, and No_drain (Figure 3B).

**Figure 4:**
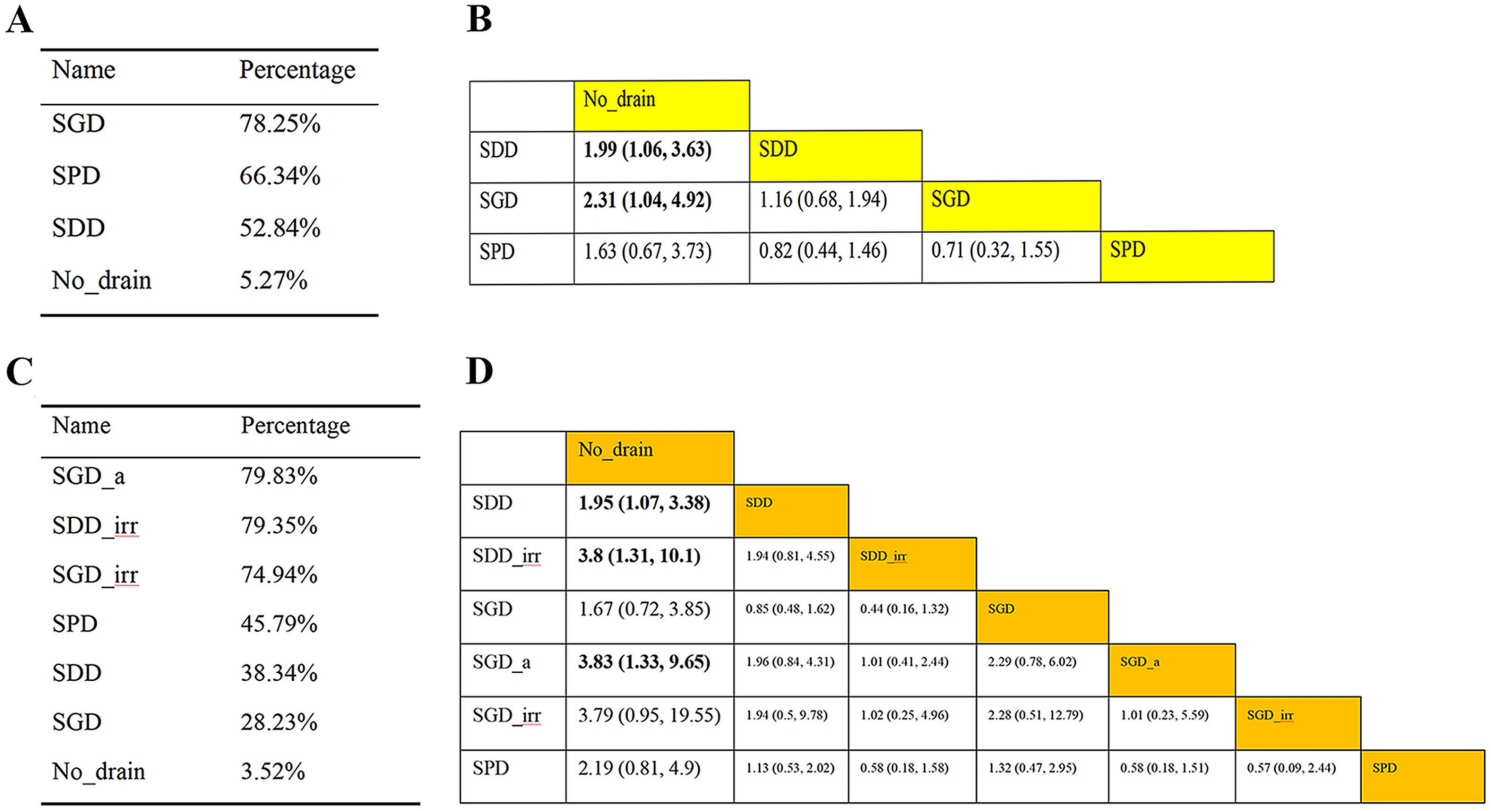
Recurrence analysis of patients with CSDH. **(A)** SUCRA values based on Classification I; **(B)** league table based on Classification I; **(C)** SUCRA values based on Classification II; **(D)** league table based on Classification II. SGD_a, subgaleal active drainage; SDD_irr, subdural irrigation drain; SGD_irr, subgaleal irrigation drain; SPD, subperiosteal drain; SDD, subdural drain; SGD, subgaleal drain; CSDH, chronic subdural hematoma.

The results of Classification I are presented in Figures 4A,B. Based on the league table, compared to No_drain, both SDD [risk ratio (RR) = 0.50, 95% CrI: 0.28–0.94] and SGD (RR = 0.43, 95% CrI: 0.20–0.96) were significantly associated with the recurrence rate. Based on the SUCRA, SGD (78.25%) exhibited the best performance among all interventions.

The results of Classification II are presented in Figures 4C,D. Based on the league table, compared to No_drain, SDD (RR = 0.51, 95% CrI: 0.30–0.93), SDD_irr (RR = 0.26, 95% CrI: 0.10–0.76), and SGD_a (RR = 0.26, 95% CrI: 0.10–0.75) were all significantly associated with a reduced risk of recurrence. Based on the SUCRA, SGD_a (79.83%) exhibited the best performance among all interventions.

#### 3.4.2 Death

**Figure 5:**
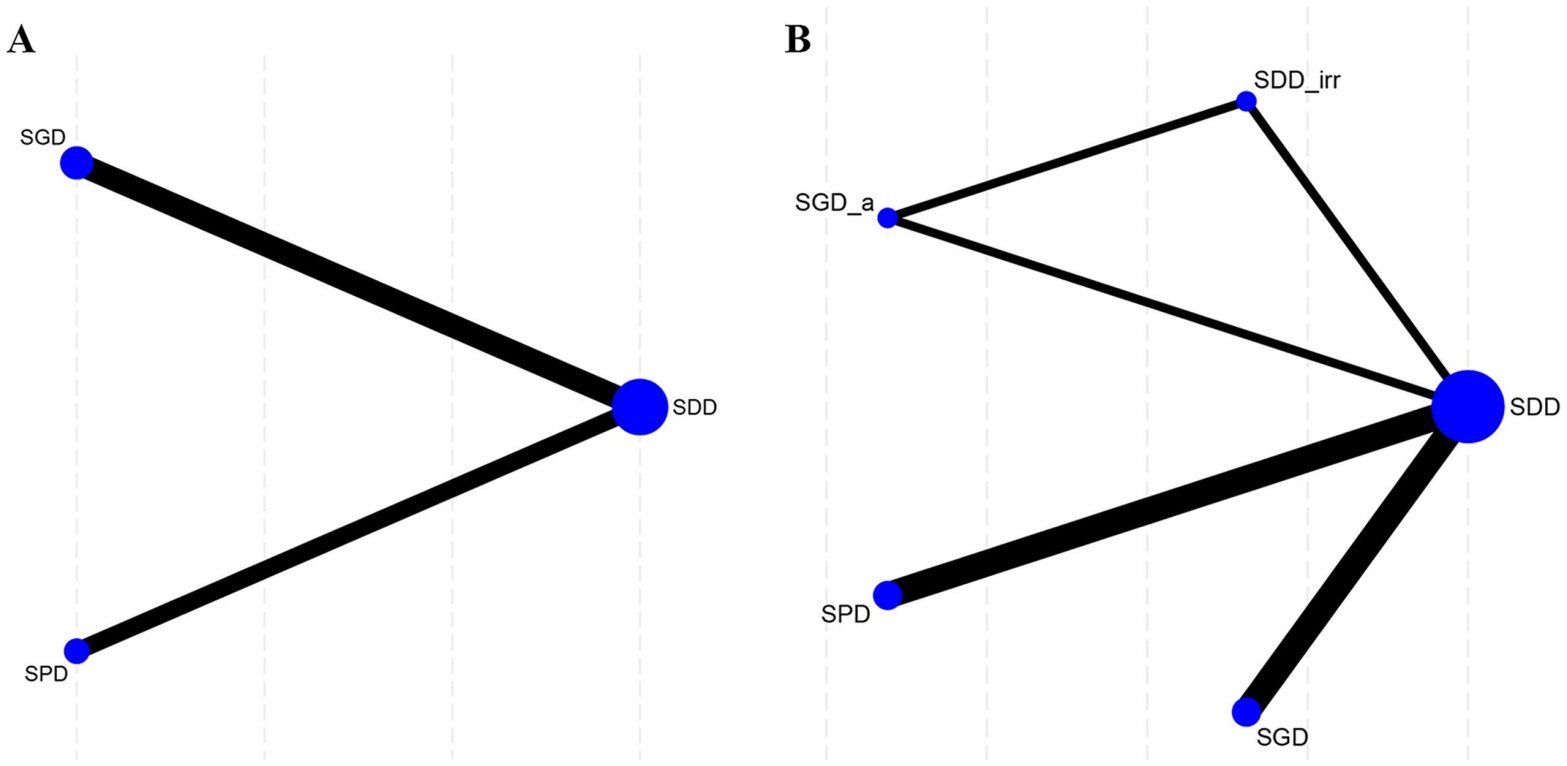
Network plots comparing SDD, SPD, and SGD in relation to mortality and recurrence. **(A)** Mortality in Classification I; **(B)** Mortality in Classification II. SDD, subdural drain; SPD, subperiosteal drain; SGD, subgaleal drain; SDD_irr, subdural irrigation drain; SGD_a, subgaleal active drainage.

Eight articles explored the impact of drain placement location on mortality in patients with CSDH. Classification I is presented in Figure 5A, which compares SDD, SPD, and SGD; direct associations were noted between each item. Classification II is presented in Figure 5B, which compares SDD, SDD_irr, SPD, SGD, and SGD_a.

**Figure 6:**
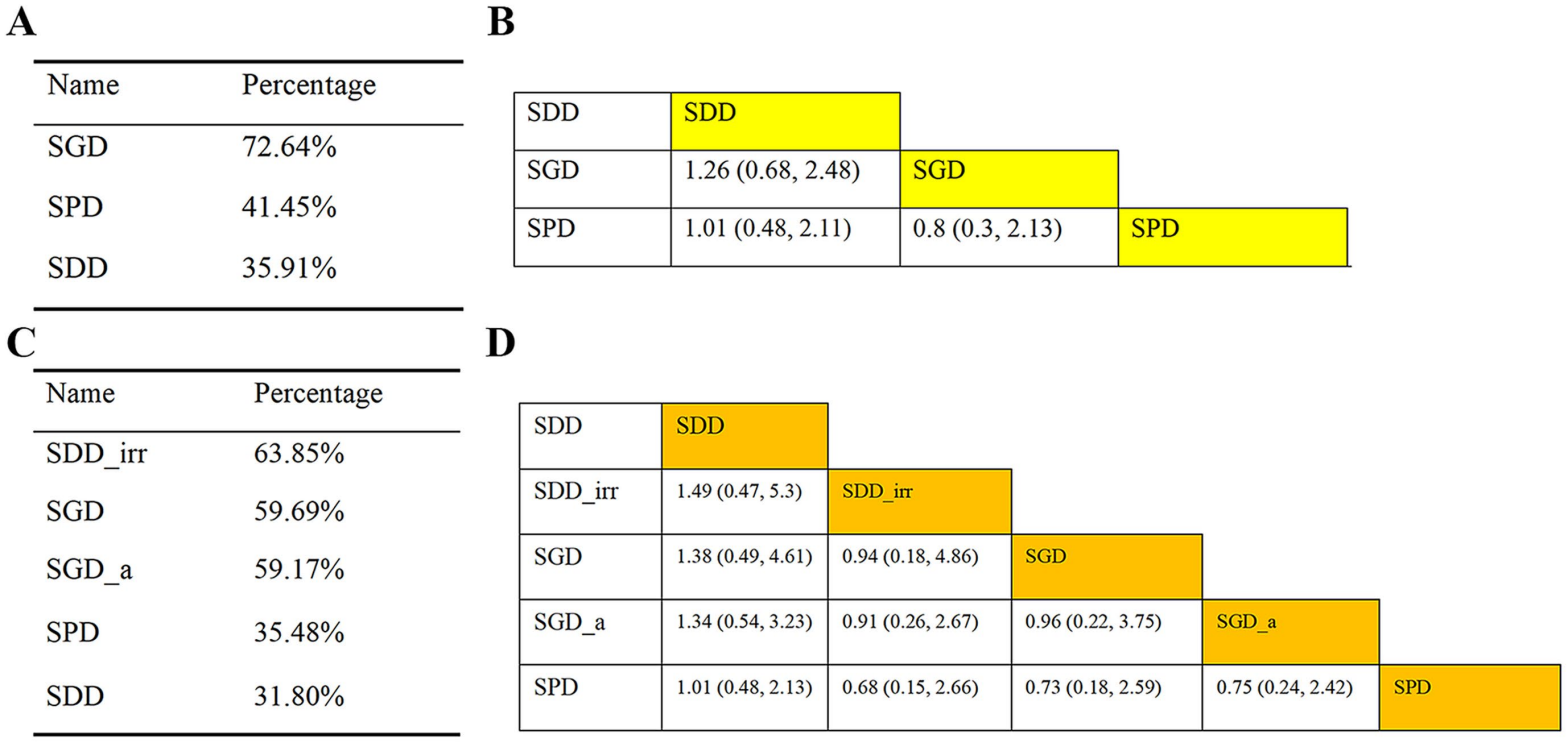
Mortality analysis of patients with CSDH. **(A)** SUCRA values based on Classification I; **(B)** league table based on Classification I; **(C)** SUCRA values based on Classification II; **(D)** league table based on Classification II. SDD, subdural drain; SPD, subperiosteal drain; SGD, subgaleal drain; SDD_irr, subdural irrigation drain; SGD_a, subgaleal active drainage; CSDH, chronic subdural hematoma.

Outcomes of mortality were analyzed using league tables and SUCRA values under both classification systems (Figure 6). According to the league tables (Figures 6B,D), no significant differences in mortality were observed among the interventions within either Classification I or II. The SUCRA rankings, however, identified the most effective interventions for reducing mortality. Under Classification I, SGD ranked highest (SUCRA = 72.64%, Figure 6A). Under Classification II, SDD_irr emerged as the most advantageous (SUCRA = 63.85%, Figure 6C).

#### 3.4.3 Complication

Brain parenchymal injury, a key safety indicator, occurred at a significantly higher rate in the SDD group (2.8%) than in the SGD group (0%) (*p* < 0.05). This suggested that the drainage location was associated with the risk of mechanical injury to brain tissue (13, 31, 35). This difference may be due to the increased likelihood of tearing the bridging vein when pulling the frontal lobe meninges during the drain placement in SDD (10, 30). Regarding neurologic-specific complications, the incidence of pneumocephalus was 8.5% higher in the SDD_irr group (47.4%) than in the SGD_irr group (38.9%). The incidence of seizures was 2.5% higher in the SDD_irr group (5.3%) than in the SGD_irr group (2.8%). This suggested that the modified procedure optimized the subdural aerodynamic balance (12). A multicenter randomized trial conducted by Soleman et al. (7) found that the rate of surgical infections and medical morbidity through drain placement was significantly lower in the SPD group than in the SDD group (*p* < 0.05). Results analyzed up to 1 year showed an advantage for the SPD group in terms of surgical infections and postoperative hemorrhage. However, at 6 weeks and 1 year, the SPD group had a relatively higher number of seizure episodes. Further calculations of seizure episodes and statistical tests are needed to determine whether these differences are significant.

#### 3.4.4 Publication bias

**Figure 7:**
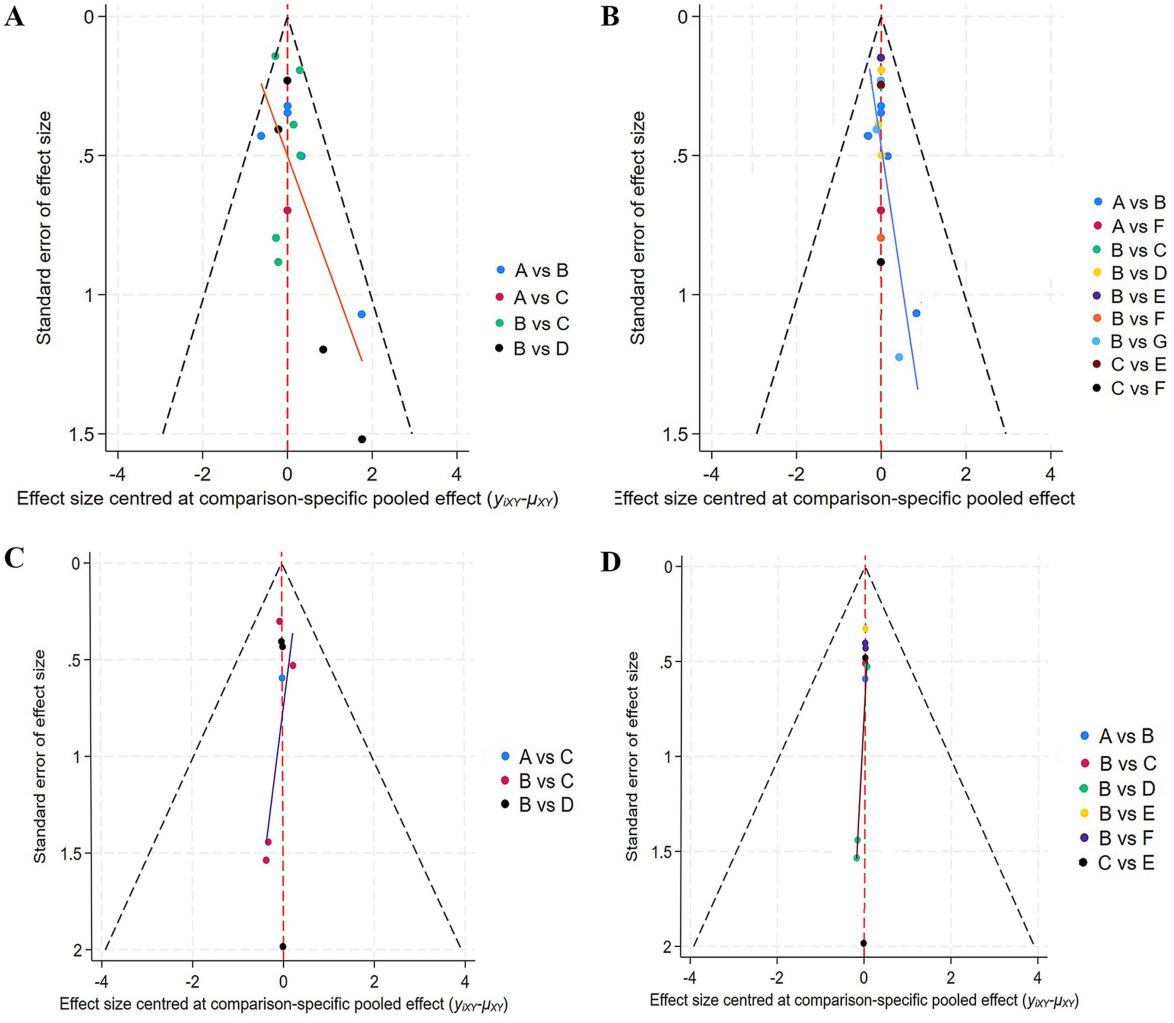
Funnel plots showing the bias for recurrence and mortality. **(A)** Recurrence in Classification I; **(B)** recurrence in Classification II; **(C)** mortality in Classification I; **(D)** mortality in Classification II.

The publication bias of included studies was systematically assessed using funnel plots (Figure 7). By plotting scatter plots with sample size on the *X*-axis and effect size on the *Y*-axis, the results demonstrated that the scatter points were distributed relatively balanced around the pooled effect size indicated by the red line. This suggested no publication bias in the included studies. Further tests based on Egger’s method revealed that the *p*-values were all greater than 0.05 for (A) Recurrence in Classification I (PEgger = 0.627); (B) Recurrence in Classification II (PEgger = 0.46); (C) Mortality in Classification I (PEgger = 0.951); (D) Mortality in Classification II (PEgger = 0.981), indicating no significant publication bias for any of the outcomes.

## 4 Discussion

The present NMA integrated 14 articles on CSDH treated with frontal or parietal burr-hole drainage. The analysis compared the effects of SDD, SPD, and SGD on postoperative recurrence and mortality. For Classification I, both league tables and SUCRA values demonstrated that SGD was advantageous in reducing recurrence and was associated with lower mortality. For Classification II, the league tables showed that SGD_a and SDD_irr were associated with lower recurrence. SUCRA values indicated that SGD_a was more effective at reducing recurrence. Regarding mortality, league tables indicated no significant differences between interventions. However, SUCRA values suggested that SDD_irr was potentially beneficial in reducing mortality.

In this NMA, it is important to distinguish between statistical and clinical heterogeneity. Our analysis revealed moderate statistical heterogeneity, as assessed by the *I*^2^ statistic. This was addressed by adopting a random-effects model. However, there was considerable clinical heterogeneity across the included studies, which may have influenced the comparative outcomes. Key sources of the heterogeneity included: (1) age distribution, as some studies enrolled both younger and older patients (≥70 years), who have distinct physiological conditions and recurrence risks; (2) surgical techniques, specifically the use of single versus double burr-holes, which may affect the completeness of hematoma evacuation; (3) duration of drainage, which ranged from 24 h to several days and can affect the rate of brain re-expansion; and (4) irrigation versus no irrigation, a variation that may influence the clearance of neomembranes and inflammatory factors. These factors could act as effect modifiers, potentially confounding direct comparison among the different drainage modalities.

According to the analysis of Classification I, numerous previous findings have fully confirmed the advantages of SGD. A 2019 study by Häni et al. (13) showed that SGD significantly reduced recurrence rates compared to SDD. Furthermore, clinical practice has revealed that the frontal burr-hole is more susceptible to damage when the drain is placed in the position of SDD. This procedure increases the risk of iatrogenic brain parenchymal injury, which may result in persistent neurological dysfunction in severe cases. This is mainly because the location of SDD is close to the cerebral cortex and bridging veins, posing a risk of direct injury to the brain parenchyma during insertion and removal of the catheter (16, 36). Compared to SDD, SGD can effectively mitigate the risk of acute bleeding caused by damage to the neomembrane from drain placement (37). You et al. (38) point out that old patients demonstrate suboptimal brain reexpansion and that the formation of postoperative pneumocephalus significantly increases the recurrence risk of CSDH. This prolongs hospital stays and wound-healing cycles and may lead to neurological deterioration. The effectiveness of SGD in preventing postoperative pneumocephalus may contribute to improved functional outcomes (39, 40).

According to the analysis of Classification II, SGD_a is highly effective in reducing the recurrence rate. Yadav et al. (41) have stated that SGD_a has the characteristics of high safety, low operational difficulty, and minimal invasiveness. Hence, it is particularly suitable for high-risk populations (37). According to the findings of other studies, SGD_a has the potential to significantly reduce the risk of brain laceration, formation of intracerebral hematoma, and seizures because it does not come into direct contact with the brain parenchyma (42). Conversely, although SDD_irr clearly reduces mortality, its applicability is limited. Studies have indicated that using SDD_irr to treat patients with coagulation disorders can lead to an increased risk of intraoperative and postoperative bleeding due to their abnormal coagulation mechanisms (43). Old and frail patients with severe underlying diseases are prone to complications such as pulmonary infections and heart failure due to poor surgical tolerance and weak postoperative recovery ability when treated with SDD_irr (43, 44). Previous studies have shown that SDD is often used routinely in clinical practice due to the anatomical characteristics of a large subdural space. However, patients’ specificity has not been adequately assessed. Based on the results of this study, risk stratification and individualized treatment are recommended. SGD_a is preferred for old patients (≥70 years of age), especially those with combined cerebral atrophy or coagulation abnormalities (INR > 1.5). This procedure demonstrates a low recurrence rate (RR = 0.26), and no secondary parenchymal brain injury is observed. SDD_irr, on the other hand, is more suitable for younger people without coagulation disorders in a low-risk population.

In this study, we first classified drainage modalities as SDD, SPD, or SGD according to anatomical location. Then, a systematic analysis was performed to assess the differences in efficacy between the locations. The results confirmed that SGD_a and SDD_irr both showed advantages in reducing the recurrence rate. These results provide an evidence-based foundation for selecting individualized clinical procedures.

However, there are certain limitations. First, the proportion of SPD patients in the sample distribution of references was relatively low, accounting for only 10% of the total sample size. Due to the imbalanced structure of samples, there is a possibility that the general applicability and accuracy of the study results may be affected. Second, the classification of interventions by anatomical location and technical method introduced additional clinical heterogeneity because the same drainage location could be combined with different surgical techniques. This should be considered when interpreting the results, as the observed effects may be confounded by these co-interventions. Third, the certainty of the evidence was rated as low to moderate due to indirect comparisons and clinical heterogeneity. Therefore, the conclusions should be interpreted with caution and should not be overgeneralized to all patient populations. Further large-scale, prospective studies with standardized surgical protocols are needed to confirm these findings and explore the interaction between clinical factors and treatment efficacy.

## 5 Conclusion

The current study indicates that SGD_a is more effective at reducing the recurrence rates of CSDH, while SDD_irr is more effective at reducing the mortality. Due to the physiological characteristics and disease features of old and high-risk populations, a comprehensive and careful assessment is necessary when applying SDD_irr clinically. Furthermore, multicenter studies with large samples and well-designed methodologies are necessary to further explore the clinical value of SGD_a and SDD_irr. The results of this study offer valuable information for implementing early interventions and developing personalized treatment plans for patients with poor prognoses.

## Data Availability

The original contributions presented in the study are included in the article/Supplementary material, further inquiries can be directed to the corresponding author.
