# Supplementary material for: Optimal drain position after evacuation of chronic subdural hematomas: a systematic review and network meta-analysis
Source: Front Neurol. 2026 May 12;17:1706424. doi: 10.3389/fneur.2026.1706424 (PMC13201130; doi:10.3389/fneur.2026.1706424)
Supplement: Supplementary file 1 [file Supplementary_file_1.docx]

**File S1** Retrieval strategy

| PUBMED | | |
| --- | --- | --- |
| SEARCH | Query | Results |
| #1 | "hematoma, subdural, chronic"[MeSH Terms] | 2266 |
| #2 | "acute subdural haematoma"[Title/Abstract] OR "acute subdural hematoma"[Title/Abstract] OR "chronic"[Title/Abstract] OR "chronic subdural haematoma"[Title/Abstract] OR "chronic subdural haematomata"[Title/Abstract] OR "chronic subdural hematoma"[Title/Abstract] OR "Chronic Subdural Hematomas"[Title/Abstract] OR "hematoma"[Title/Abstract] OR "hemorrhagic pachymeningitis"[Title/Abstract] OR "intracranial subdural haematoma"[Title/Abstract] OR "intracranial subdural hematoma"[Title/Abstract] OR "intracranial subdural hematomas"[Title/Abstract] OR "pachymeningitis haemorrhagica"[Title/Abstract] OR "subdural"[Title/Abstract] OR "subdural bleeding"[Title/Abstract] OR "subdural haematoma"[Title/Abstract] OR "subdural haemorrhage"[Title/Abstract] OR "subdural hematoma"[Title/Abstract] OR "subdural hemorrhage"[Title/Abstract] | 1687063 |
| #3 | #1 OR #2 | 1687112 |
| #4 | Drainage[MeSH Terms] | 69006 |
| #5 | drain*[Title/Abstract] OR rainage[Title/Abstract] | 177361 |
| #6 | #4 OR #5 | 211458 |
| #7 | #3 AND #6 | 18222 |

| Embase | | |
| --- | --- | --- |
| SEARCH | Query | Results |
| #1 | 'subdural hematoma'/exp OR 'subdural hematoma' | 28317 |
| #2 | 'acute subdural haematoma':ab,ti OR 'acute subdural hematoma':ab,ti OR 'chronic':ab,ti OR 'chronic subdural haematoma':ab,ti OR 'chronic subdural haematomata':ab,ti OR 'chronic subdural hematoma':ab,ti OR 'chronic subdural hematomas':ab,ti OR 'chronic subdural hematomata':ab,ti OR 'haemorrhagic pachymeningitis':ab,ti OR 'hematoma':ab,ti OR 'hemorrhagic pachymeningitis':ab,ti OR 'intracranial subdural haematoma':ab,ti OR 'intracranial subdural haematomas':ab,ti OR 'intracranial subdural haematomata':ab,ti OR 'intracranial subdural hematoma':ab,ti OR 'intracranial subdural hematomas':ab,ti OR 'intracranial subdural hematomata':ab,ti OR 'pachymeningiosis haemorrhagica interna':ab,ti OR 'pachymeningitis haemorrhagica':ab,ti OR 'subdural':ab,ti OR 'subdural bleeding':ab,ti OR 'subdural haematoma':ab,ti OR 'subdural haemorrhage':ab,ti OR 'subdural hematoma':ab,ti OR 'subdural hemorrhage':ab,ti OR 'subepidural haematoma':ab,ti OR 'subepidural hematoma':ab,ti | 2490188 |
| #3 | #1 OR #2 | 2499941 |
| #4 | 'drain*':ab,ti OR 'drainage':ab,ti | 260341 |
| #5 | #3 AND #4 | 27725 |

| Cochrane | | |
| --- | --- | --- |
| SEARCH | Query | Results |
| #1 | MeSH descriptor: [Drainage] explode all trees | 4018 |
| #2 | MeSH descriptor: [Hematoma, Subdural, Chronic] explode all trees | 192 |
| #3 | ('acute subdural haematoma):ti,ab,kw OR (acute subdural hematoma):ti,ab,kw OR (chronic):ti,ab,kw OR (chronic subdural haematoma):ti,ab,kw OR (chronic subdural haematomata):ti,ab,kw OR (chronic subdural hematoma):ti,ab,kw OR (Chronic Subdural Hematomas):ti,ab,kw OR (chronic subdural hematomata):ti,ab,kw OR (haemorrhagic pachymeningitis):ti,ab,kw OR (hematoma):ti,ab,kw OR (hemorrhagic pachymeningitis):ti,ab,kw OR (intracranial subdural haematoma):ti,ab,kw OR (intracranial subdural haematomas):ti,ab,kw OR (intracranial subdural haematomata):ti,ab,kw OR (intracranial subdural hematoma):ti,ab,kw OR (intracranial subdural hematomas):ti,ab,kw OR (intracranial subdural hematomata):ti,ab,kw OR (pachymeningiosis haemorrhagica interna):ti,ab,kw OR (pachymeningitis haemorrhagica):ti,ab,kw OR (subdural):ti,ab,kw OR (subdural bleeding):ti,ab,kw OR (subdural haematoma):ti,ab,kw OR (subdural haemorrhage):ti,ab,kw OR (subdural hematoma):ti,ab,kw OR (subdural hemorrhage):ti,ab,kw OR (subepidural haematoma):ti,ab,kw OR (subepidural hematoma'):ti,ab,kw | 214867 |
| #4 | #2 OR #3 | 214867 |
| #5 | (drain*):ti,ab,kw OR (Drainage):ti,ab,kw | 17006 |
| #6 | #1 OR #5 | 18351 |
| #7 | #4 AND #6 | 2246 |
|  | To exclude Cochrane Reviews (59 records) and retain only Trials (2187 records) | 2187 |

| Web of Science | | |
| --- | --- | --- |
| SEARCH | Query | Results |
| #1 | TS=(drain* OR Drainage) | 337965 |
| #2 | TS=(“acute subdural haematoma“ OR ”acute subdural hematoma“ OR ”chronic“ OR ”chronic subdural haematoma“ OR ”chronic subdural haematomata“ OR ”chronic subdural hematoma“ OR ”Chronic Subdural Hematomas“ OR ”chronic subdural hematomata“ OR ”haemorrhagic pachymeningitis“ OR ”hematoma“ OR ”hemorrhagic pachymeningitis“ OR ”intracranial subdural haematoma“ OR ”intracranial subdural haematomas“ OR ”intracranial subdural haematomata“ OR ”intracranial subdural hematoma“ OR ”intracranial subdural hematomas“ OR ”intracranial subdural hematomata“ OR ”pachymeningiosis haemorrhagica interna“ OR ”pachymeningitis haemorrhagica“ OR ”subdural“ OR ”subdural bleeding“ OR ”subdural haematoma“ OR ”subdural haemorrhage“ OR ”subdural hematoma“ OR ”subdural hemorrhage“ OR ”subepidural haematoma“ OR ”subepidural hematoma”) | 1776832 |
| #3 | #1 AND #2 | 14875 |
