# Supplementary material for: Optimal drain position after evacuation of chronic subdural hematomas: a systematic review and network meta-analysis
Source: Front Neurol. 2026 May 12;17:1706424. doi: 10.3389/fneur.2026.1706424 (PMC13201130; doi:10.3389/fneur.2026.1706424)
Supplement: Supplementary file 2 [file Table_1.docx]

**Table S1** Main characteristics of the studies included in the network meta-analysis.

| **Author (Year)** | **Nationality** | **Type** | **Title** | **Ntotal** | **Treatment** | **N** | **Age (Years)** | **Gender (f/m)** | |
| --- | --- | --- | --- | --- | --- | --- | --- | --- | --- |
| Sukru Oral 2015 | Turkey | Cohort | Comparison of subgaleal and subdural closed drainage system in the surgical treatment of chronic subdural hematoma | 74 | SGD_irr | 36  38 | 68.1±14.4 | 8/28 | |
|  |  |  |  |  | SDD_irr | 38 | 66.1±13.7 | 9/29 | |
| David Yuen Chung Chan2016 | Hong Kong | Cohort | Use of subdural drainage for chronic subdural haematoma? A 4-year multi-centre observational study of 302 cases | 302 | SDD | 149 | 76 (46–93) | 33/149 | |
|  |  |  |  |  | No_drain | 153 | 74 (42–95) | 30/153 | |
| AdriAn ng Wei Chih 2017 | Malaysia | Cohort | Subperiosteal Drainage versus Subdural Drainage in the Management of Chronic Subdural Hematoma (A Comparative Study) | 60 | SDD | 30 | 70 | 10/20 | |
|  |  |  |  |  | SPD | 30 | 68 | 9/21 | |
| Kristin Sjvik 2016 | Denmark | Cohort | Assessment of drainage techniques for evacuation of chronic subdural hematoma: a consecutive population-based comparative cohort study | 1260 | SDD_irr | 166 | 73 | 55/111 | |
|  |  |  |  |  | SDD | 330 | 74 | 81/249 | |
|  |  |  |  |  | SGD_a | 764 | 74 | 246/518 | |
| Laurence Johann Glancz 2019 | British | Cohort | Does Drain Position and Duration Influence Outcomes in Patients Undergoing Burr-Hole Evacuation of Chronic Subdural Hematoma? Lessons from a UK Multicenter Prospective Cohort Study | 577 | SDD | 533 | 78 (98-85) | 183/394 | |
|  |  |  |  |  | SGD | 44 |  |  |  |
| Jehuda Soleman 2019 | Switzerland | RCT | Subperiosteal vs. Subdural Drainage After Burr-Hole Drainage of Chronic Subdural Hematoma: A Randomized Clinical Trial (cSDH-Drain-Trial) | 220 | SDD | 100 | 81.0 (74.0-85.0) | 32/68 | |
|  |  |  |  |  | SPD | 120 | 78.0 (70.7-83.2) | 39/81 | |
| Yong Woo Shim 2019 | Korea | Cohort | Burr Hole Drainage versus Small Craniotomy of Chronic Subdural Hematomas | 75 | SDD | 60 | 74.5 (67–90) | 16/44 | |
|  |  |  |  |  | Surgery | 15 | 73.2 (65–83) | 2/13 | |
| John J.Y. Zhang 2019 | British | Cohort | Outcomes of Subdural Versus Subperiosteal Drainage After Burr-Hole Evacuation of Chronic Subdural Hematoma: A Multicenter Cohort Study | 570 | SDD | 329 | 71 (61–79) | | 78/251 |
|  |  |  |  |  | SPD | 241 | 70 (62–80) | | 70/171 |
| Laurence Johann Glancz 2020 | British | Cohort | Drains result in greater reduction of subdural width and midline shift in burr hole evacuation of chronic subdural haematoma | 317 | SDD | 272 | 78 (67–85) | | 88/184 |
|  |  |  |  |  | No_drain | 45 | 71 (56–81) | | 14/31 |
| Levin Häni 2020 | Switzerland | Cohort | Subdural versus subgaleal drainage for chronic subdural hematomas: a post hoc analysis of the TOSCAN trial | 361 | SDD | 214 | 72.58 ± 11.02 | | 75/139 |
|  |  |  |  |  | SGD | 147 | 74.83 ± 11.02 | | 42/105 |
| Kolakoth Pathoumthong 2021 | Thailand | RCT | Comparative study of subdural drainage (SDD) versus subperiosteal drainage (SPD) in treating patients with chronic subdural hematoma (CSDH) | 42 | SDD | 21 | 65.33 | | 9/12 |
|  |  |  |  |  | SPD | 21 |  |  | 5/16 |
| Utku OZGEN 2022 | Turkey | Cohort | A Comparison of Subgaleal Active Drainage and Subdural Passive Drainage and an Analysis of Factors Affecting Chronic Subdural Hematoma Outcomes | 87 | No_drain | 52 | 72 ± 14.08 | | 19/33 |
|  |  |  |  |  | SDD | 20 | 74 ± 9.5 | | 7/13 |
|  |  |  |  |  | SGD_irr | 15 | 70 ± 16.2 | | 3/12 |
| Sheng Zhang 2022 | China | RCT | Solve the post-operative subdural pneumatosis of chronic subdural hematoma: A novel active bone hole drainage system | 79 | SDD | 49 | 76.00 (67.00–82.00) | | 10/39 |
|  |  |  |  |  | Surgery | 30 | 69.00 (64.25–78.25) | | 4/26 |
| Sandra Li 2024 | Australia | Cohort | Subdural Versus Subgaleal Drain Placement After Minicraniotomy for Chronic Subdural Hematoma | 137 | SDD | 103 | 72.3 ± 12 | | 30/73 |
|  |  |  |  |  | SGD | 34 | 67.6 ± 13 | | 12/22 |

SDD: Subdural drainage, SDD_irr: Subdural irrigation drainage, SPD: Subperiosteal drainage, SGD: Subgaleal drainage, SGD_irr: Subgaleal irrigation drainage, SGD_a: Subgaleal irrigation active.
