# Supplementary material for: Optimal drain position after evacuation of chronic subdural hematomas: a systematic review and network meta-analysis
Source: Front Neurol. 2026 May 12;17:1706424. doi: 10.3389/fneur.2026.1706424 (PMC13201130; doi:10.3389/fneur.2026.1706424)
Supplement: Supplementary file 3 [file Table_2.docx]

**Table S2** Methodological Quality of Prospective Case-control Studies Based on NOS.

| **Included studies** | **Study population selection** | **Comparability** | **Exposure or results** | **Levels** |
| --- | --- | --- | --- | --- |
| Sukru Oral 2015 | ☆☆☆ | ☆☆ | ☆☆ | 7☆ |
| David Yuen Chung Chan 2016 | ☆☆ | ☆☆ | ☆☆ | 6☆ |
| AdriAn ng Wei Chih 2017 | ☆☆ | ☆☆ | ☆☆ | 6☆ |
| Kristin Sjvik 2016 | ☆☆ | ☆☆ | ☆☆ | 6☆ |
| Laurence Johann Glancz 2019 | ☆☆☆ | ☆☆ | ☆☆ | 7☆ |
| Yong Woo Shim 2019 | ☆☆☆ | ☆☆ | ☆☆ | 7☆ |
| John J.Y. Zhang 2019 | ☆☆ | ☆☆ | ☆☆ | 6☆ |
| Laurence Johann Glancz 2020 | ☆☆ | ☆☆ | ☆☆ | 6☆ |
| Levin Häni 2020 | ☆☆☆ | ☆☆ | ☆☆ | 7☆ |
| Utku OZGEN 2022 | ☆☆☆ | ☆☆ | ☆☆ | 7☆ |
| Sandra Li 2024 | ☆☆☆ | ☆☆ | ☆☆ | 7☆ |

NOS: Newcastle-Ottawa Scale.
